# Quantitative assessment of urban wetland dynamics using high spatial resolution satellite imagery between 2000 and 2013

**DOI:** 10.1038/s41598-018-25823-9

**Published:** 2018-05-09

**Authors:** Tangao Hu, Jiahong Liu, Gang Zheng, Yao Li, Bin Xie

**Affiliations:** 1 Zhejiang Provincial Key Laboratory of Urban Wetlands and Regional Change, Hangzhou Normal University, Hangzhou, 311121 China; 2 The State Key Laboratory of Satellite Ocean Environment Dynamics, Second Institute of Oceanography, State Oceanic Administration, Hangzhou, 310012 China

## Abstract

Accurate and timely information describing urban wetland resources and their changes over time, especially in rapidly urbanizing areas, is becoming more important. We applied an object-based image analysis and nearest neighbour classifier to map and monitor changes in land use/cover using multi-temporal high spatial resolution satellite imagery in an urban wetland area (Hangzhou Xixi Wetland) from 2000, 2005, 2007, 2009 and 2013. The overall eight-class classification accuracies averaged 84.47% for the five years. The maps showed that between 2000 and 2013 the amount of non-wetland (urban) area increased by approximately 100%. Herbaceous (32.22%), forest (29.57%) and pond (23.85%) are the main land-cover types that changed to non-wetland, followed by cropland (6.97%), marsh (4.04%) and river (3.35%). In addition, the maps of change patterns showed that urban wetland loss is mainly distributed west and southeast of the study area due to real estate development, and the greatest loss of urban wetlands occurred from 2007 to 2013. The results demonstrate the advantages of using multi-temporal high spatial resolution satellite imagery to provide an accurate, economical means to map and analyse changes in land use/cover over time and the ability to use the results as inputs to urban wetland management and policy decisions.

## Introduction

Wetlands are the most productive ecosystems in nature and important environments for humans^1^. Wetlands are known as the “kidneys of the earth” because of their important hydrological features and element cycles, and they can also be considered as “biological supermarkets” because of their large food webs and rich biological diversity^2,3^. Wetlands have far-reaching influences on the urban environment, including on the hydrological cycle, flood control, shoreline protection, climatic regulation, landscape building, natural species protection, and ecosystems service functions^4–7^. However, as human activity has continuously increased over the past hundred years, many wetlands have been lost, and the remaining wetland areas have substantially decreased, which has caused a large loss of natural habitats^8,9^. Recently, due to the progress of society and the rapid development of modern industry, land reclamation, water pollution and excessive deforestation have become increasingly serious issues, especially for urban wetlands. With the acceleration of urbanization, a large number of wetlands disappeared^10^. Monitoring urban wetlands and detecting their changes over specified time periods are necessary to ensure wetlands protection and optimal use^11^.

Satellite remote sensing provides a considerable opportunity to monitor land use and land cover (LULC) and detect changes because of the rapid, synoptic and repetitive capabilities of remote sensing^12^. Multi-source satellite images provide efficient information on the temporal trends and spatial distributions of urban areas that is needed to understand, model, and project land changes^13^. Specifically, moderate-resolution multispectral sensors (e.g., Landsat, SPOT, and ASTER) have been successfully used for studying the extent of flooding and affected land cover^14^, detecting the presence and composition of wetlands in heterogeneous landscapes^15^, and monitoring invasive plant species^16^. However, few studies have focused on the detection of small wetlands and ponds as small as 0.2 ha that are often important critical habitats. Sensors with 10 to 30-m spatial resolution typically require a minimum of 9 pure pixels (0.9 ha) to identify a feature^17^. Because of mixed pixels, the smaller wetlands are often missed^18^. At a local scale, the small wetlands are crucial to the stability of ecosystems, particularly for the maintenance of biodiversity^19^. Very high spatial resolution multispectral sensors (e.g., QuickBird and IKONOS) provide fine spatial resolution to capture smaller wetlands^20^, but the within-class spectral variance will be increased using these sensors. Therefore, in order to solve this problem, an appropriate classification algorithm must be employed^21,22^.

Recently, object-based image analysis (OBIA) has been applied more frequently for image classification and change detection in wetland systems^23,24^. Object-based approaches consider landscapes as aggregations of meaningful objects corresponding to ground entities and patches of surface cover^25^. In addition to being applied to extract features in urban and rural landscapes, OBIA techniques help provide detailed classifications of natural plant communities that are represented in wetland areas^26,27^. Previous studies have found that the OBIA approach performed better than the pixel-based approach for classifying urban areas and mapping LULC and LULC changes^20,28^. Fournier *et al*. reviewed mapping methods for wetland application and identified the OBIA approach as the most appropriate due to its ability to solve the spatial heterogeneity of wetlands^19,29^. OBIA approaches have also been successfully used in wetlands research to classify major wetland cover types^25^ wetland vegetation species^27^ and map wetlands in both natural and human landscapes^19^.

There are various methods for using satellites to determine wetland change in urban environments^30–33^. These methods can be broadly grouped into two general types, change enhancement methods and ‘from-to’ change information extraction methods. The former method (for example, image differencing) does not explicitly identify the types of land that have changed and only provides change/no change information and perhaps the relative magnitude of the change. The latter method, the post-classification approach, is one of the most commonly used and effective techniques^11^. Post-classification comparison methods use separate classifications for images acquired at different times to produce difference maps from which ‘from-to’ change information can be generated^34^. In addition, ‘from-to’ quantitative information about the type of LULC changes can be gained from a cross-tabulated change matrix^33^.

The Xixi Wetland is located in downtown Hangzhou, a city in China known for its rapid economic development and tourism, and it is a Ramsar Site^11^. However, with economic development and the expansion of the city, this area of wetlands has decreased rapidly. Its wetland functions have gradually become degraded, environmental pollution has substantially increased, and wetlands protection has become more difficult. The Xixi Wetland has faced combined pressures from natural and human factors. For protection purposes, quantitative wetland change detection and a driving factors analysis are essential to better understanding the relationship between anthropic activities and natural wetland systems.

This paper describes the methods and results of classifications and post-classification change detection of multi-temporal high spatial resolution satellite imagery of the Xixi Wetland for 2000, 2005, 2007, 2009, and 2013. The main objectives of this study are as follows: (1) develop a change detection method to map and monitor urban wetland changes between 2000 and 2013; (2) assess the accuracy of multi-temporal classifications; (3) quantify the area of change and spatial distribution change for urban wetlands; and (4) analyse urban wetland loss patterns and relate them to driving factors.

## Materials

### Study area

**Figure 1:**
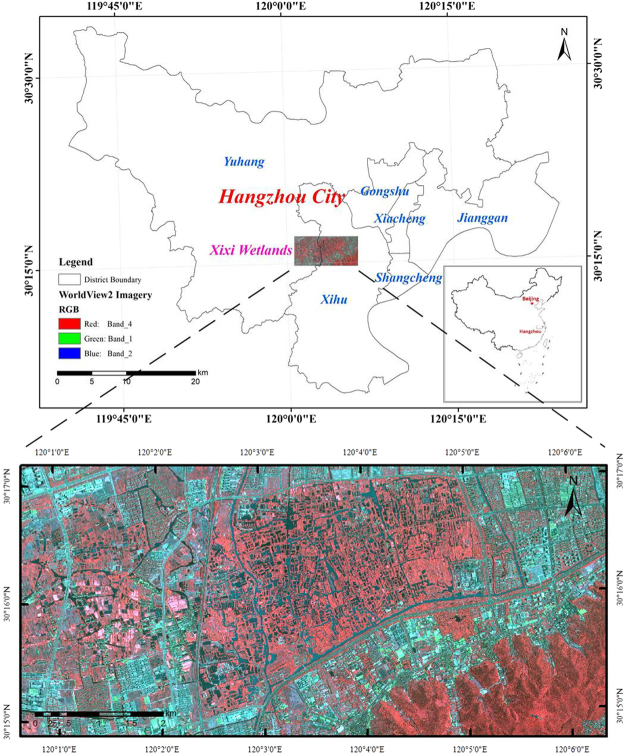
Location of the study area. The aerial photograph was produced by high spatial resolution satellite imagery: WorldView2 data on May 17, 2012. The data were acquired from Siwei Worldview Technology (Beijing) Co., Ltd. For more information about Siwei Worldview Technology (Beijing) Co., Ltd., please visit www.siweidg.com. This figure was created using ArcGIS^®^ software by ESRI. ArcGIS 10.2 and ArcMap 10.2, which are the intellectual property of ESRI and are used here in under license. For more information about ESRI software, please visit www.esri.com.

The Xixi Wetland, which is known as the “kidneys of the earth”, was selected as the study area (Fig. 1). The wetland complex is located west of Hangzhou City between 30°15′–30°17′N and 120°1′–120°6′E. The wetland previously covered an area as large as 60 km^2^, but it has shrunk to 11 km^2^ because of urbanization in Hangzhou over the past 30 years. The wetland mainly contains fish ponds, rivers, and vegetation communities. In addition, the wetland also contains centralized villages and a few scattered areas of houses and trees^35^. The circled squares in the map of the Xixi Wetland show the rapidly developing economic zone. The area of wetlands has gradually been reduced due to industrialization and urbanization, including building construction, reclamation, and pollution. The resources in the Xixi Wetland face combined pressures from natural and socio-economic factors that must be resolved. Therefore, the Hangzhou Xixi National Wetland Park was constructed in 2005 to protect the Xixi Wetland. In 2009, it was successfully included in the “List of Wetlands of International Importance (the Ramsar List)”^36^.

### Data acquisition and processing

#### High spatial resolution satellite imagery

To detect LULC changes, images that represent several stages within the same season are preferable. However, it was difficult to find time-series of high spatial resolution satellite images that were well matched within the same season for several reasons but primarily due to the weather conditions in this area. Five cloud-free images were acquired in January 2000, December 2005, January 2007, December 2009, and December 2013, covering the whole Xixi Wetland area, and these images were selected under the constraints of limited suitable images in the archives (Table 1). All images were acquired from the Siwei Worldview Technology (Beijing) Co., Ltd. (www.siweidg.com). All images were provided in the Universal Transvers Mercator projection (UTM Zone 50 N) and WGS84 datum. These were careful georeferenced to a root mean squared error within 2 pixels using a 1^st^-order polynomial transformation method. Finally, they were clipped to the same extent.

**Table 1:** Satellite imagery information.

Sensor	Acquisition date	Spatial resolution	Spectral resolution
IKONOS	20 January 2000	Panchromatic: 0.82 m Multispectral: 3.28 m	Blue: 445–516 nm Green: 506–595 nm Red: 632–698 nm Near infrared: 757–853 nm
IKONOS	19 December 2005	—	—
QuickBird	28 January 2007	Panchromatic: 0.73 m Multispectral: 2.90 m	Blue: 450–520 nm Green: 520–600 nm Red: 630–690 nm Near infrared: 760–900 nm
QuickBird	30 December 2009	—	—
WorldView2	29 December 2013	Panchromatic: 0.52 m Multispectral: 2.40 m	Blue: 450–510 nm Green: 510–580 nm Red: 630–690 nm Near infrared: 770–895 nm

#### Reference data

Reference data were developed for each of the five years and then randomly divided for classifier training and accuracy assessment. The reference data for 2013 were a field verified set of reference sites collected in the early spring of 2012. This data set was created by collecting cover type information for a stratified random sample of 160 points with 20 points per level III class. The strata were from a previous classification result of 2000 IKONOS imagery. At each sample point, a field computer with ArcPAD^®^ 10.2 by ESRI and GPS were used to digitize a polygon of the area from the 2013 cover type identified, with other cover types near the sampled point. Reference data for the 2000 were derived from interpretation of very high resolution (VHR) aerial photographs acquired in the summer of 2000 that were produced by the Zhejiang Administration of Surveying Mapping and Geoinformation. Reference data for 2005, 2007 and 2009 were derived from the interpretation of historical Google Earth imagery, and the data were acquired at a similar time. The reference data included approximately 200 polygons for each year; approximately 70% of the data were used for training and 30% for the accuracy assessment.

## Methods

### Classification system

Currently, many international organizations and scientists have developed wetland classification systems based on different research perspectives^37^. The Land cover classification system developed by Anderson *et al*.^34^ and the Ramsar Convention database^10^ are used to define classification system used in this. The Ramsar definition of wetlands is broad, including not only vegetated wetlands but also lakes, coral reefs, and even underground caves. Therefore, the classification system is an open-ended, three-level hierarchical system organized by classes and types to provide robust information on wetlands (Table 2). At the first level, all land types are defined as urban wetlands or non-wetland areas according to the Ramsar definition. At the next level, urban wetland is divided into vegetation and water, and non-wetland mainly contains urban areas. At the third level, vegetation is classified by the dominant vegetation type: forest, herbaceous, cropland, or marsh vegetation; water is classified as river or pond; urban is classified as building or road.

**Table 2:** LULC classification system.

Level I	Level II	Level III	Description
Urban wetland	Vegetation	Forest	Deciduous forest land, evergreen forest land, mixed forest land, orchards, groves
		Herbaceous	Land where vegetation is dominated by a mix of grasses, grass-like plants, shrubs or bush; either naturally occurring or modified (e.g., roadside vegetation, meadows, mixed composition short vegetation upland)
		Cropland	Crop fields, pasture, and bare fields
		Marsh	A wetland that is dominated by herbaceous rather than woody plant species. At the edges of lakes and streams, where they form a transition between the aquatic and terrestrial ecosystems.
	Water	River	Such as stream, creek, brook, rivulet, and rill
		Pond	A body of standing water, either natural or artificial, that is usually smaller than a lake
Non-wetland	Urban	Building	Areas of intensive use where much of the land is covered by man-made structures (e.g., residential, commercial, industrial, utility)
		Road	Such as parkways, avenues, freeways, interstates, highways, and tertiary local roads.

### Feature extraction

Feature extraction uses an object-based approach to classify imagery, where an object (also called a segment) is a group of pixels with similar spectral, spatial, and/or texture attributes. Traditional classification methods are pixel-based, meaning that the spectral information in each pixel is used to classify the imagery. With high-resolution panchromatic or multispectral imagery, an object-based method offers more flexibility in terms of the types of features that can be extracted. In this study, the object-based approach in the ENVI^®^ 5.3.1 by EVIS (Exelis Visual Information Solutions, Inc., Broomfield, CO, USA) was used for wetland information extraction. This approach was mainly a two-step process, segmentation and classification^38^. In addition, to improve the accuracy of classification results, a visual interpretation based on high resolution images from Google Earth imagery was used for post-classification.

#### Feature development

Six input layers were used for the image segmentation process, including four multispectral layers (blue, green, red, and near-infrared), a normalized difference vegetation index (NDVI) layer, and a standard deviation texture layer^19^. The NDVI is a simple graphical indicator that can be used to assess whether the pixel contains live green vegetation or not, and can also be used to separate water from dry land and delineate wetland boundaries^19^. The NDVI layer was calculated from the red and near-infrared bands of the satellite images. Texture refers to the spatial variation of image tone as a function of scale and can reveal differences between classes in a digital image. Texture can also be created from satellite imagery without additional data. According to the method proposed by Mui* et al*., we created a standard deviation texture layer using a 3 × 3 pixel moving filter window^19^. Finally, all input layers were weighted equally in the segmentation process^19^.

#### Image segmentation

Segmentation is the process of partitioning an image into objects by grouping neighbouring pixels with common values. The objects in the image ideally correspond to real-world features. Effective segmentation ensures that classification results are more accurate. An edge-based segmentation algorithm, which computes a gradient map based on a user defined scale level, was used in this study. The scale level was chosen so that adjacent pixels with similar characteristics could be grouped. Since the aim of the study was to classify the wetland into several levels of cover types, a scale level of 25% was chosen after several iterations. We decided not to merge the images after segmentation to avoid any loss of information. Texture attributes were computed for each kernel, and 3 was selected as the default value. Finally, watershed transformation was applied to the modified gradient map to produce the final segmentation results^39^.

#### Image classification

Training samples were selected using high spatial resolution satellite imagery and ground truth data obtained through field surveys. A minimum of 30 training samples were chosen for each class^19^. Approximately 20 samples were directly imported from field datasets and approximately 10 samples were added by interpretation. Different classification methods have their own merits and can achieve different classification results. While the nearest neighbour, or k-NN approach, is among the simplest of all machine learning algorithms, it performs as well as more complicated classifiers such as support vector machines (SVM) or neural net. In this study a nearest neighbour classifier (non-parametric classifier) was selected to classify images according to the defined classification system. The k-NN approach is a very intuitive method that classifies unlabelled examples based on their similarity to examples in the training set. The separation distance between classes was calculated based on the mean feature values of the pixels in each object (calculated from the input layers). The k-NN process involved selecting training samples, comparing sample attributes, and refining training samples until a satisfactory result was achieved^19^. After the k-NN method runs, each segment is assigned the class with the highest-class confidence value.

### Change detection

Following the classification results from the individual years, a multi-date post-classification comparison change detection algorithm was used to determine the changes in land cover for four intervals, 2000 to 2005, 2005 to 2007, 2007 to 2009, and 2009 to 2013. This process is the most common approach for change detection and has been successfully used in several land-use changes studies^34,40^. The post-classification approach provides “from-to” change information, and the type of landscape transformations that have occurred can be easily calculated and mapped. A change detection map of “from-to” change information was derived for each of the five classification maps.

### Accuracy assessment

An evaluation of the quality of a map derived from remote sensing data is important not only for verifying the quality of the map and its fitness for a specific purpose but also for understanding the errors in a map and their likely implications. Many methods have been used for evaluating map accuracy. The most popular method includes the confusion matrix, used for maps derived from most types of classification methods^10^. In this study, overall accuracy and kappa coefficient were used for accuracy assessment^19^. The kappa coefficient is a measure of chance-corrected agreement between the actual land-cover classes and the classified land-cover classes in remote sensing. In this study, the kappa coefficient was calculated using the following equation:

1 $$k=\frac{{\rm{N}}{\sum }_{i=1}^{r}{n}_{ii}-{\sum }_{i=1}^{r}({n}_{i+}\times {n}_{+i})}{{N}^{2}-{\sum }_{i=1}^{r}({n}_{i+}\times {n}_{+i})}$$

where *r* represents the number of rows in the matrix, *n*_*ii*_ represents the number of observations in row *i* and column *i*, *n*_+*i*_ and *n*_*i*+_ are the marginal totals of row *i*, column *i*, respectively, and *N* represents the total number of observations in the matrix.

For most purposes, kappa values are interpreted as follows: (1) values ≥0.75 indicate excellent agreement beyond chance, (2) values ≥0.4 to <0.75 indicate fair to good agreement beyond chance, and (3) values <0.4 indicate poor agreement beyond chance^10^.

## Results

### Accuracy assessment of the map

A quantitative accuracy assessment was conducted to evaluate the accuracies of the maps. A total of 60 random samples were selected for the accuracy assessment. Sample selection was based upon very high resolution aerial photographs and Google Earth imagery. The samples were divided into verification data and training data. Ground truth data was collected in February 2012. The overall accuracies were calculated as 91.53% for 2000, 87.69% for 2005, 89.84% for 2007, 84.47% for 2009, and 88.24% for 2013. Kappa coefficients were calculated as 0.82 for 2000, 0.71 for 2005, 0.73 for 2007, 0.69 for 2009, and 0.72 for 2013. These results suggest that all five maps show an acceptable range of agreement with the reference data used for the accuracy assessment.

### Classification and change maps

**Figure 2:**
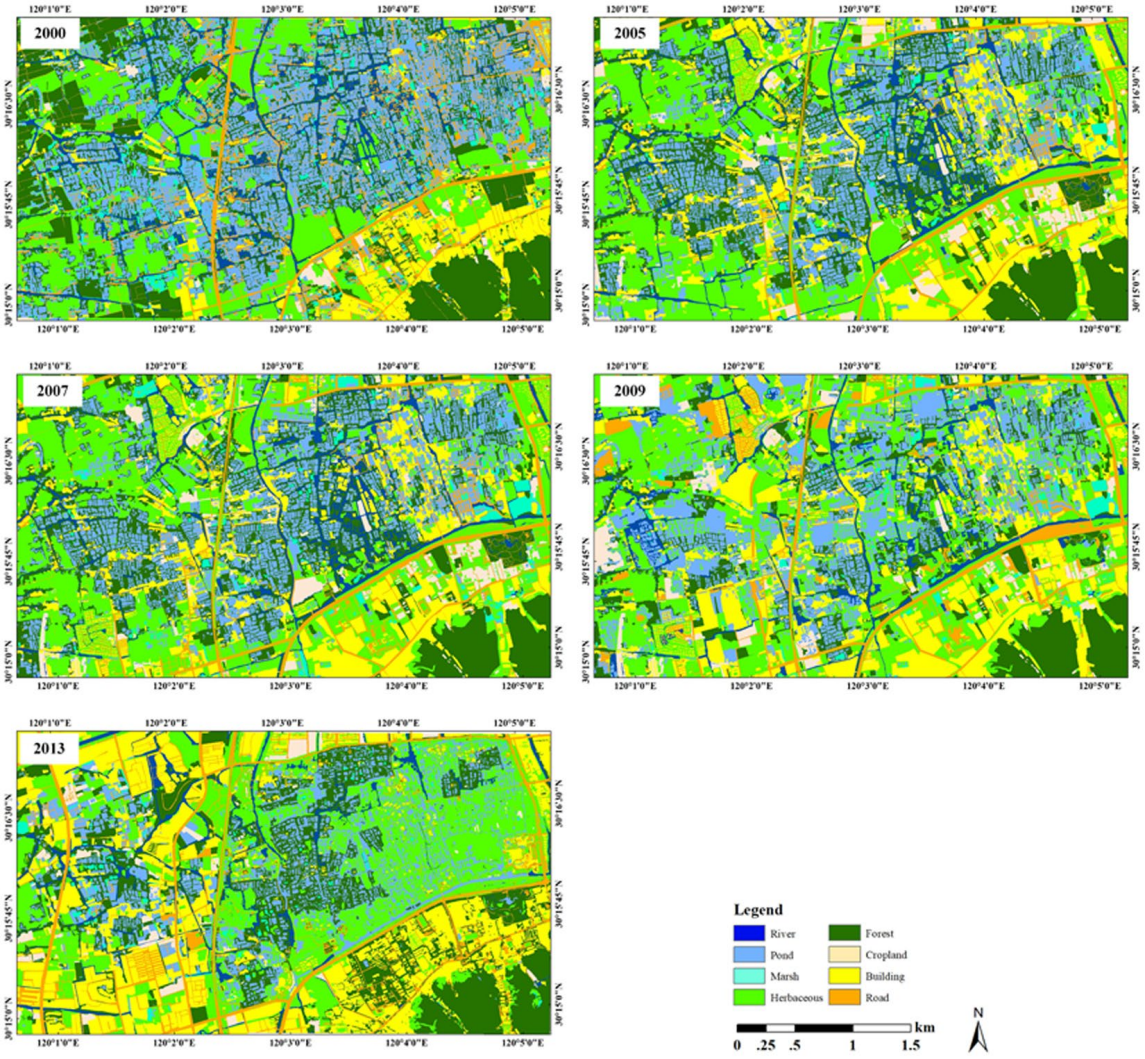
Land-cover classification maps from 2000 to 2013 for the Xixi Wetlands. The maps were produced by high spatial resolution satellite imagery: IKONOS data on January 20, 2000 and December 19, 2005, QuickBird data on January 28, 2007 and December 30, 2009, and WorldView2 data on December 29, 2013. The images were acquired from Siwei Worldview Technology (Beijing) Co., Ltd. For more information about Siwei Worldview Technology (Beijing) Co., Ltd., please visit www.siweidg.com. This figure was created using ENVI^®^ software by EVIS. ENVI 5.3.1 is the intellectual property of EVIS and is used here under license. For more information about ENVI software, please visit http://www.harrisgeospatial.com/SoftwareTechnology/ENVI.aspx.

**Figure 3:**
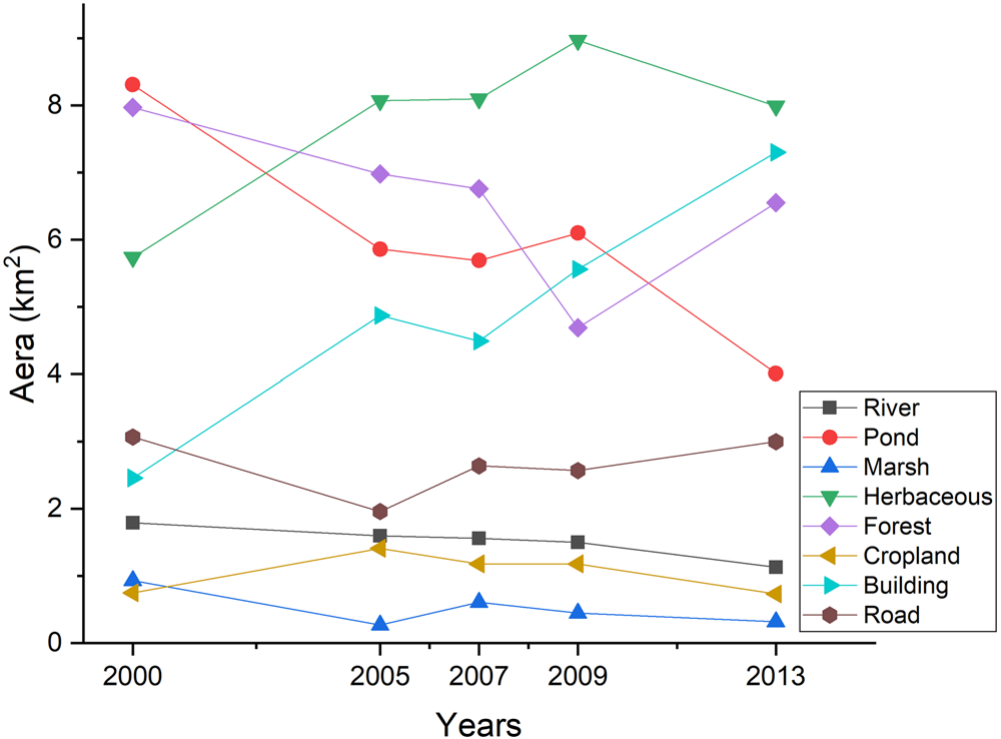
The change statistics for five years (2000, 2005, 2007, 2009 and 2013). This figure was created using Origin^®^ 2018 by OriginLab Corporation. Origin is an industry-leading scientific graphing and data analysis software and is used here under license. For more information about Origin software, please visit www.originlab.com.

Classification maps were generated for all five years (Fig. 2), and the individual class areas and change statistics for the five years are summarized in Fig. 3. From 2000 to 2013, the building land-cover type increased the most in terms of area (by approximately 4.84 km^2^), and the herbaceous land-cover type increased by approximately 2.25 km^2^, while the pond land-cover type decreased the most in terms of area (by approximately 4.3 km^2^), followed by forest (decreased by approximately 1.42 km^2^), river (decreased by approximately 0.66 km^2^), and marsh (decreased by approximately 0.61 km^2^). In terms of the percent change in area, the non-wetland (urban) land-cover type increased by approximately 100% from 2000 to 2013, while wetlands (vegetation and water) decreased by approximately 20%, and pond decreased the most (by approximately 50%).

To further evaluate the results of the LULC conversions, matrices of LULC changes from 2000 to 2005, 2005 to 2007, 2007 to 2009, 2009 to 2013 and 2000 to 2013 were calculated (Tables 3 and 4). In the tables, unchanged pixels are located along the major diagonal of the matrix. From 2000 to 2005, pond (approximately 1.17 km^2^) and forest (approximately 1.03 km^2^) were the main land-cover types that changed to non-wetland areas. From 2005 to 2007, herbaceous (approximately 0.62 km^2^) and cropland (approximately 0.35 km^2^) were the main land-cover types that changed to non-wetland areas. From 2007 to 2009, herbaceous (approximately 1.47 km^2^) and forest (approximately 0.4 km^2^) were the main land-cover types that changed to non-wetland areas. Compared to the last period, the rate of change increased. From 2009 to 2013, herbaceous (approximately 2.94 km^2^) and pond (approximately 0.77 km^2^) were the main land-cover types that changed to non-wetland areas. The greatest increase in non-wetland area occurred from 2007 to 2013. Based on the matrices of the LULC changes, herbaceous, forest, and pond are the areas most likely to be converted to new buildings and roads. In addition, we found that the main change areas are distributed around the Xixi National Wetland Park because the areas in the park are strictly protected by law^36^. To analyse how many wetlands were converted to the non-wetland land-cover type, we calculated the specific change types shown in Table 5. The results show that herbaceous (32.22%), forest (29.57%) and pond (23.85%) are the main land-cover types that changed to non-wetland, followed by cropland (6.97%), marsh (4.04%) and river (3.35%).

**Table 3:** Matrices of LULC and changes (km^2^) from 2000 to 2005, 2005 to 2007, 2007 to 2009 and 2009 to 2013.

2005	2000								2005 Total
	River	Pond	Marsh	Herbaceous	Forest	Cropland	Building	Road	
River	0.68	0.38	0.07	0.15	0.2	0.02	0.02	0.09	1.6
Pond	0.32	4.43	0.17	0.21	0.45	0.05	0.03	0.21	5.86
Marsh	0.01	0.15	0.01	0.04	0.03	0	0.01	0.02	0.27
Herbaceous	0.02	0.78	0.19	3.36	2.22	0.29	0.49	0.55	8.07
Forest	0.32	1.24	0.21	0.71	3.85	0.13	0.09	0.43	6.98
Cropland	0.03	0.17	0.05	0.53	0.19	0.08	0.25	0.11	1.41
Building	0.18	0.9	0.19	0.49	0.71	0.16	1.36	0.88	4.87
Road	0.05	0.27	0.05	0.25	0.32	0.04	0.2	0.77	1.96
2000 Total	1.79	8.31	0.93	5.74	7.97	0.75	2.46	3.07	31.03
**2007**	**2005**								**2007 Total**
River	1.46	0.01	0	0.04	0.02	0.01	0	0.02	1.56
Pond	0.01	5.51	0	0.07	0.06	0.01	0.02	0.01	5.69
Marsh	0	0.02	0.25	0.08	0.04	0.04	0.18	0	0.61
Herbaceous	0.07	0.1	0.01	6.83	0.43	0.1	0.49	0.06	8.1
Forest	0.02	0.07	0	0.26	6.28	0.05	0.05	0.01	6.76
Cropland	0.01	0.05	0	0.18	0.02	0.85	0.06	0	1.18
Building	0.01	0.04	0	0.3	0.04	0.25	3.84	0.02	4.49
Road	0.02	0.06	0	0.32	0.09	0.1	0.22	1.84	2.64
2005 Total	1.6	5.86	0.27	8.07	6.98	1.41	4.87	1.96	31.03
**2009**	**2007**								**2009 Total**
River	0.96	0.04	0	0.11	0.32	0.02	0.02	0.03	1.5
Pond	0.09	5.03	0.01	0.2	0.64	0.02	0.05	0.07	6.1
Marsh	0	0.01	0.4	0	0.01	0	0	0.02	0.45
Herbaceous	0.35	0.24	0.13	5.48	1.48	0.18	0.25	0.87	8.97
Forest	0.11	0.15	0.02	0.4	3.87	0.01	0.05	0.1	4.69
Cropland	0	0.01	0	0.45	0.03	0.63	0.02	0.04	1.18
Building	0	0.13	0.03	0.74	0.22	0.23	4.04	0.16	5.56
Road	0.05	0.08	0.02	0.73	0.18	0.09	0.05	1.37	2.57
2007 Total	1.56	5.69	0.61	8.1	6.76	1.18	4.49	2.64	31.03
**2013**	**2009**								**2013 Total**
River	0.44	0.21	0	0.28	0.08	0.01	0.06	0.05	1.13
Pond	0.37	2.4	0.06	0.68	0.26	0.03	0.12	0.09	4.01
Marsh	0.01	0.16	0	0.06	0.02	0.01	0.02	0.03	0.32
Herbaceous	0.25	1.28	0.11	3.06	1.1	0.41	1.15	0.62	7.99
Forest	0.24	1.17	0.06	1.67	2.38	0.1	0.64	0.3	6.55
Cropland	0.01	0.1	0.01	0.29	0.11	0.07	0.11	0.04	0.73
Building	0.12	0.6	0.14	2.09	0.56	0.41	2.91	0.48	7.3
Road	0.06	0.17	0.07	0.85	0.19	0.15	0.55	0.96	3
2009 Total	1.5	6.1	0.45	8.97	4.69	1.18	5.56	2.57	31.03

**Table 4:** Matrices of LULC and changes (km^2^) from 2000 to 2013.

2013	2000								2013 Total
	River	Pond	Marsh	Herbaceous	Forest	Cropland	Building	Road	
River	0.48	0.26	0.04	0.1	0.14	0.01	0.02	0.07	1.13
Pond	0.4	2.54	0.11	0.35	0.43	0.01	0.02	0.14	4.01
Marsh	0.01	0.15	0.02	0.04	0.08	0.01	0	0.02	0.32
Herbaceous	0.35	2.07	0.23	1.98	2.03	0.15	0.25	0.92	7.99
Forest	0.28	1.44	0.21	0.64	3.02	0.06	0.38	0.52	6.55
Cropland	0.03	0.14	0.03	0.32	0.15	0.01	0.01	0.04	0.73
Building	0.17	1.28	0.23	1.65	1.63	0.22	1.5	0.64	7.3
Road	0.07	0.43	0.06	0.66	0.49	0.28	0.27	0.73	3
2000 Total	1.79	8.31	0.93	5.74	7.97	0.75	2.46	3.07	31.03

**Table 5:** Specific change types from 2000 to 2013.

Change type	Specific change types	Area (km^2^)	Percent (%)
Wetland to non-wetland	River to non-wetland (urban)	0.24	3.35
	Pond to non-wetland (urban)	1.71	23.85
	Marsh to non-wetland (urban)	0.29	4.04
	Herbaceous to non-wetland (urban)	2.31	32.22
	Forest to non-wetland (urban)	2.12	29.57
	Cropland to non-wetland (urban)	0.50	6.97

### Analysis of change patterns

**Figure 4:**
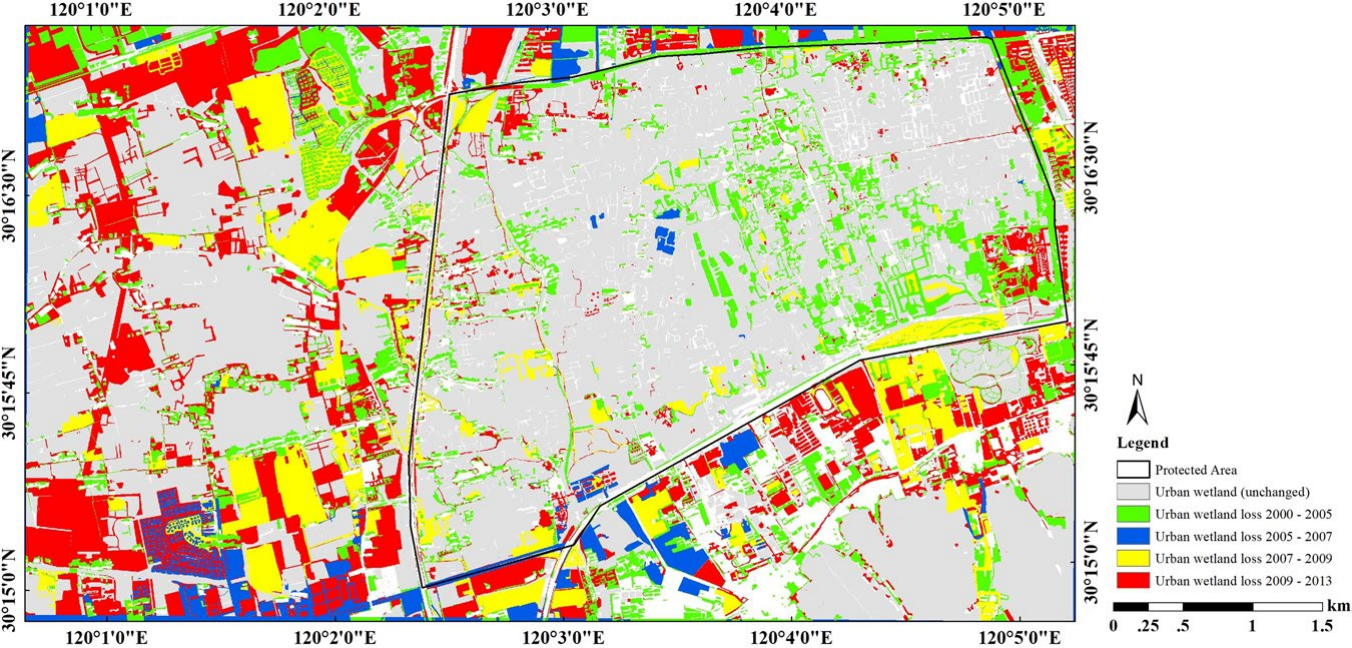
Urban wetland loss from 2000 to 2013. This figure was created using ArcGIS^®^ software by ESRI. ArcGIS 10.2 and ArcMap 10.2 are the intellectual property of ESRI and are used here under license. For more information about ESRI software, please visit www.esri.com.

Although similar statistics could be generated for other units such as county, township, or census tract, etc., the above change statistics shed minimal light on the question of where LULC changes are occurring. However, by constructing a change detection map (Fig. 4), the advantages of satellite remote sensing in spatially disaggregating change statistics can be more fully appreciated. Figure 4 shows the process of urban wetland loss in the study area from 2000 to 2005 (approximately 1.81 km^2^/year), 2005 to 2007 (approximately 0.61 km^2^/year), 2007 to 2009 (approximately 1.26 km^2^/year) and 2009 to 2013 (approximately 1.35 km^2^/year). Therefore, urban wetlands declined most from 2000 to 2005, followed by 2009 to 2013 and 2007 to 2009. The unchanged areas from 2000 to 2013 were about 15.4 km^2^. In addition, after 2005, in the protected area, urban wetland areas only slightly declined, because the local government established Xixi National Wetland Park to protect Xixi Wetlands in 2005. However, the regions outside of the protected area declined rapidly, especially after 2007. Based on Fig. 4, the increase in urban areas is mainly distributed west and southeast of the study area, and the greatest increase occurred from 2007 to 2013. In summary, information from satellite remote sensing can play a significant role in quantifying and understanding the nature of changes in LULC and where the changes are occurring. This information is essential to planning for urban growth and development.

## Discussion and Conclusions

The LULC changes in the Xixi Wetland are affected by many factors including natural and human factors. Natural factors, which often operate on large spatial and temporal scales, combine with landscape effects to control changes in wetlands at a large environmental scale. However, human-related factors operate at short time scales and are important driving forces that affect changes in wetlands, especially in urban wetlands. In order to protect urban wetlands, the Xixi National Wetland Park was constructed in 2005 and the local government established several laws to protect wetlands in the park^35^. However, these laws only protect the wetlands inside the park, and the wetlands outside the park are not protected. The local economy developed rapidly because of tourist attractions, especially after 2007^41^. Therefore, while the region inside of the protected area remains stable, the adjacent area declined rapidly. As shown in Fig. 4, most of the wetland changes occurred west and southeast of the study area, and patterns emerged that highlight urbanization activity (e.g., real estate exploitation and population growth). The Xixi Wetland is a primary example of the nature-based tourism attractions in the southern part of China, and it is the first national urban wetland area in China to receive five A grades for tourist attractions. Because of the comfortable environment, many real estate enterprises are investing in the area and developing and building houses around the Xixi National Wetland Park^41^. Many houses have even been built directly on herbaceous, forest, and pond land-cover types. Many rivers and ponds have been filled, and landscape connections have been altered by the buildings and roads found throughout the wetlands. Therefore, large areas of urban wetlands have been replaced by built-up land due to urbanization^42^.

High spatial resolution satellite images covering several years and an object-based classification method were used to interpret the study area, classify different types of wetlands and analyse spatial and subordinate relationships based on the reference data. This approach guarantees the consistency of the wetlands classification system and allows for a rapid acquisition of historical data for the wetlands. Therefore, this method plays an important role in analysing the variation rules and driving mechanisms of urban wetlands. However, there are some uncertainties and errors in the change detection results, which are mainly due to variations in the data and the classification accuracy. For example, the remote sensing images were obtained over five years, from 2000 to 2013, and between January and December. Next, some of the land-cover types such as forest and herbaceous are easily confused in terms of their spectral characteristics, which can affect the classification. Moreover, training samples of every LULC type will affect the classification accuracy.
